# Aerial-trained deep learning networks for surveying cetaceans from satellite imagery

**DOI:** 10.1371/journal.pone.0212532

**Published:** 2019-10-01

**Authors:** Alex Borowicz, Hieu Le, Grant Humphries, Georg Nehls, Caroline Höschle, Vladislav Kosarev, Heather J. Lynch

**Affiliations:** 1 Department of Ecology & Evolution, Stony Brook University, Stony Brook, New York, United States of America; 2 Institute for Advanced Computational Science, Stony Brook University, Stony Brook, New York, United States of America; 3 Department of Computer Science, Stony Brook University, Stony Brook, New York, United States of America; 4 HiDef Aerial Surveying Ltd., Cleator Moor, Cumbria, United Kingdom; 5 BioConsult SH GmbH & Co. KG, Husum, Germany; Politechnika Krakowska im Tadeusza Kosciuszki, POLAND

## Abstract

Most cetacean species are wide-ranging and highly mobile, creating significant challenges for researchers by limiting the scope of data that can be collected and leaving large areas un-surveyed. Aerial surveys have proven an effective way to locate and study cetacean movements but are costly and limited in spatial extent. Here we present a semi-automated pipeline for whale detection from very high-resolution (sub-meter) satellite imagery that makes use of a convolutional neural network (CNN). We trained ResNet, and DenseNet CNNs using down-scaled aerial imagery and tested each model on 31 cm-resolution imagery obtained from the WorldView-3 sensor. Satellite imagery was tiled and the trained algorithms were used to classify whether or not a tile was likely to contain a whale. Our best model correctly classified 100% of tiles with whales, and 94% of tiles containing only water. All model architectures performed well, with learning rate controlling performance more than architecture. While the resolution of commercially-available satellite imagery continues to make whale identification a challenging problem, our approach provides the means to efficiently eliminate areas without whales and, in doing so, greatly accelerates ocean surveys for large cetaceans.

## Introduction

There is tremendous interest in understanding if and how cetacean populations are recovering following the cessation of intense commercial whaling, yet their extensive ranges and high level of mobility combine with the challenges of research at sea to leave them poorly studied in many regions. While many cetacean species favor shelf edges and other zones of deep-water upwelling across ocean basins [1–4], most cetacean research is focused on coastal areas where populations may be concentrated at key times of the year and are logistically easier to survey. Far less work has been done to understand patterns of cetacean habitat use along distant continental shelf regions, and limited cetacean surveys in deep-water habitat may skew our understanding about preferred habitat [5]. While data loggers and transponders have been employed for many species to track movements far outside the range of direct observation and provide data with high spatial and temporal resolution [6–10], the cost of these devices often drastically limits the number of animals that might be tracked.

Broad-scale or basin-scale surveys are exceedingly challenging and costly, regardless of modality. Between 1975 and 2005, only 25% of the world’s oceans were surveyed for cetaceans, with a high proportion of surveys falling inside the territorial waters of the United States [5,11]. Basin-scale surveys are unavoidably multi-year efforts in which a region is surveyed in parts over consecutive years or involve extensive mark-recapture studies [12–14]. Another approach has been to combine data from heterogeneous sources such as aerial and ship surveys (e.g., [15]), strandings, and whaling data [16]. However, these methods are expensive and the results are difficult to interpret due to large data gaps. New methods that complement existing tools and address these challenges of scale are required. While remote sensing has been used for decades to track coarse-grained changes in the environment (e.g., sea ice, land cover, urban development), the use of satellite imagery to directly survey animals is much more recent and hinges on the use of very high-resolution (sub-meter) imagery that can capture individual animals on the landscape (e.g., [17–22]). While the promise of direct surveys of wildlife from space is an exciting frontier for wildlife biology, the challenges in identifying animals, which are almost always rare and usually only a few pixels in size, remain formidable. In addition to the challenges inherent to classification, the volumes of high-resolution imagery that must be annotated for a comprehensive survey are enormous and require advances in computing, storage, and cyberinfrastructure.

Cetaceans remain a challenging taxon of study given their frequently broad ranges and marine life-history, but their size makes them an attractive target for the use of imagery-based surveys. Previous efforts to locate whales using high-resolution imagery [23–25] have been largely successful yet face challenges in bringing the process to broader spatial or temporal scales given the time required for analysis [25]. Fretwell et al. [24] successfully identified Southern Right whales (*Eubalaena glacialis*) near Peninsula Valdés, Argentina both manually and using supervised and un-supervised classification algorithms in WorldView-2 satellite imagery. This satellite sensor provides 8 spectral bands and a panchromatic band with a maximum resolution of 46 cm per pixel on-nadir. More recently, WorldView-3 imagery has become available, providing a maximum resolution of 31 cm per pixel on-nadir. Cubaynes et al. [25] found that the spectral response of whales declines above the visible red band (630–690 nm). Because both manual and automated methods rely on only the visible bands, automated algorithms are easily validated by visual inspection.

Ocean basins are large, and the potential habitat of cetaceans may encompass vast areas, especially during times of migration. Without knowing where cetaceans are, far more imagery must be considered than can reasonably be annotated manually, particularly if surveys are going to be repeated with any regularity. With this increase in data volume, the only practical solution becomes a workflow that involves a high level of automation to accomplish the otherwise tedious task of manually examining millions of pixels constituting hundreds or thousands of square kilometers. Although contemporary machine learning algorithms have been in use for 20 years or more, their application to ecological datasets have only become commonplace in the past 5–10 years [26]. Machine learning applications involving computer vision are only now gaining traction as a means of managing large volumes of image data that are tedious to analyze manually, such as camera traps, aerial imagery, or time-lapse photography [27]. Such algorithms can automate the process of classifying individual features in images (e.g., [28–29]) and counting or estimating abundance (e.g., [30–31]). Driven by commercial applications, machine learning methods have progressed rapidly in the past decade, with particular interest being paid to deep-learning methods. Deep-learning algorithms have shown promise in the field of ecology, with applications in acoustic signal detection [32], behavioral predictions [33], and camera trap classification [34], and have been adopted or proposed in numerous other fields such as medicine [35] and traffic management (e.g. [36]).

We present a cetacean survey method, employing a convolutional neural network (CNN) to automate much of the satellite imagery interpretation. Our goal in this initial pilot study was not to develop a fully-automated method, but to identify images with a high probability of containing a whale and thereby minimize the labor required for expert annotation. Here we describe an initial pipeline for whale detection that makes substantial advances toward a fully-automated detection system up to and including the global scale.

## Methods

### Imagery

**Fig 1:**
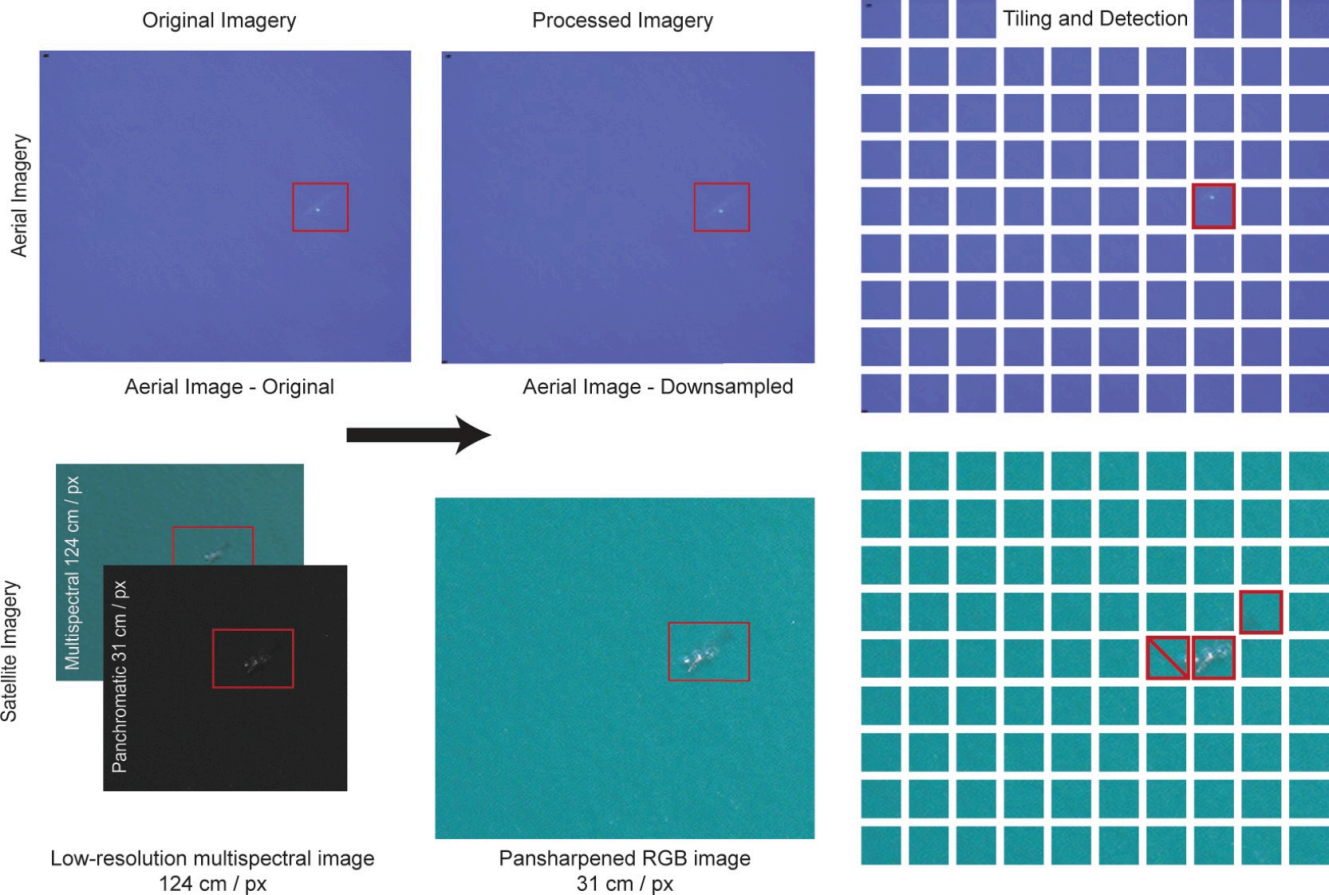
The automated workflow. Aerial imagery (above) is down-sampled, tiled, and then used to train the model. Satellite imagery (below) is pansharpened and tiled before the model can detect whales. Satellite imagery published under a CC BY license, with permission from the DigitalGlobe Foundation, original copyright 2014.

We pooled aerial imagery extracted from high-resolution video footage captured over various water bodies surrounding northern Europe and the United Kingdom by HiDef Aerial Surveying Ltd. to create a training set of whale and water images (Fig 1). The native resolution of the aerial imagery was approximately 2 cm per pixel ground-sample distance, which we down-sampled using a bilinear resampling function in ImageJ [37] to match the 31 cm resolution of Worldview-3 imagery. During the aerial survey, the aircraft flies at 549 m above sea level with a speed of 222 km/h and captures multiple image frames of the same whale using four cameras. The two inner cameras cover a 129 m wide strip and the outer cameras a 143 m wide strip; they are separated by a gap of about 20 m totaling to an effective transect width of 544 m [38]. We elected to retain these duplicate images, as each frame captures the whale at a slightly different angle or in a different body position and is therefore valuable in building the training data set. In total, we had 190 aerial images representing 17 individual minke whales (*Balaenoptera acutorostrata*).

All satellite imagery was collected at a resolution of 31 cm per pixel (on-nadir) from DigitalGlobe's Worldview-3 sensor (Digital Globe, Westminster, Colorado; S1 Table). This sensor records imagery in a variety of spectral bands in the visible and near-infrared range. Multi-spectral bands have a lower resolution (124 cm / pixel) than the panchromatic band, which incorporates a broad swath of the visible spectrum and is collected at 31 cm per pixel. To obtain very high-resolution multi-spectral imagery, we pansharpened the lower resolution multi-spectral bands using the higher resolution panchromatic band using the Gram-Schmidt algorithm implemented in ENVI (Exelis Visual Information Solutions, Boulder, Colorado). While pansharpening was conducted using all the available bands, we used only the red (630–690 nm), green (510–580 nm), and blue (450–510 nm) bands to approximate the RGB image captured during aerial survey.

**Fig 2:**
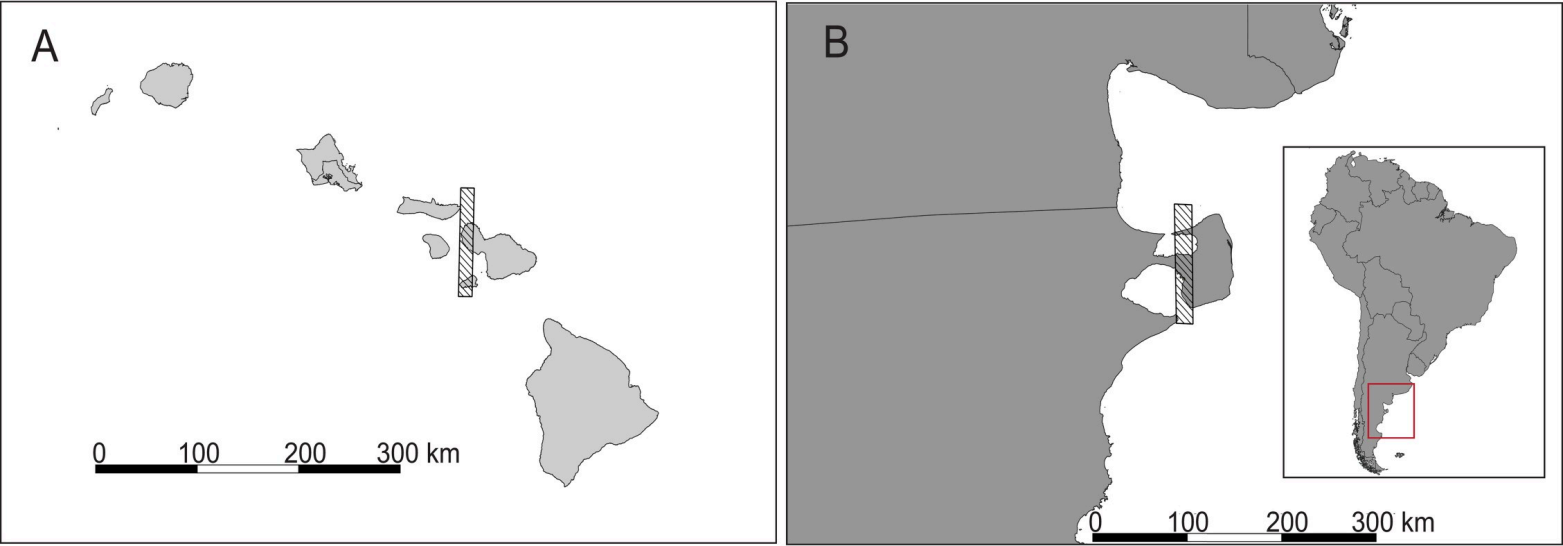
Locations of satellite imagery used. Maui, Hawaii (A) and Peninsula Valdés, Argentina (B) [39].

Given that we required satellite imagery that definitely included whales, we surveyed known whale hotspots using Google Earth Pro and located imagery that contained visible Southern Right whales (*Eubalaena australis*) from Peninsula Valdés, Argentina and Humpback whales (*Megaptera novaeangliae*) from Maui, Hawaii (Fig 2). We also acquired cloud-free imagery of these regions based on times of the year where whales would be very likely to be present and active at the surface (S1 Table). We excluded portions of the acquired imagery in which sea conditions prevented manual detection of whales.

### Processing

To prepare imagery for use in model training, we split each image into small 32 × 32-pixel tiles each measuring 98.4 m^2^ in area (Fig 1; S1 File). Different models require different input sizes and as such, we enlarged the 32 × 32-pixel tiles to 224 × 224 pixels (S2 File) For both aerial and satellite imagery, tiles were manually separated into whale and water classes. We chose subsets of each satellite image for testing but retained all aerial water tiles for training, resulting in 40,416 aerial tiles (water: n = 39,726; whale: n = 690) and 40,516 satellite tiles (water: n = 40,474; whale: n = 42). Of the water satellite tiles, we randomly selected a subset (n = 1,390) to reduce testing time.

We selectively removed some aerial tiles prior to creating validation folds. These images contained a miniscule portion of a whale that was recognizable to a human observer only when put into context with the surrounding tiles. Given that these small whales were better captured by other neighboring tiles, we removed them from both training and testing to avoid confusion. Where whales were cleanly bisected by the border between tiles, such that at least 20% of the whale remained in the tile, we retained these images. The same procedure was adopted in with the satellite imagery to avoid false-negatives for whales that would already be captured by the model in another tile, reducing the number of satellite whale tiles used for testing from 42 to 32 (Fig 1).

### Deep learning with convolutional neural networks

CNNs are extensions of traditional neural networks that work by summarizing patterns in images across many “hidden” layers through ‘convolutions’ (i.e., complex data transformations, see [7, 40]). Neural networks, the basis for CNNs, operate by simulating how neurons transmit information through the central nervous system [41–42]. Information is input at a commencing node, which then passes information to several other nodes, which pass on to more nodes, until the output is translated at a terminal node. Each node adds some function to decode the information passed into the commencing node. In CNNs, nodes are organized into “hidden” layers, where each layer applies a different image transformation (or convolution) to information from the previous layer. Transformations could include tasks such as stretching, skewing, splitting, or changing the contrast of images. During training, the training images–or images containing only the target classes, whales and water–are fed to the model so that it can ‘learn,’ i.e. adjust the network’s parameters to minimize the differences between the network’s outputs and the correct labels. The model, once converged, can be validated using a test image subset that was withheld from the training process. This form of machine learning minimizes the need for manually designing a rule-based classification scheme, but limits the interpretability of the model, which acts as a black box. Deep learning is a rapidly-evolving field with new architectures regularly outstripping the performance of previous methods.

### Model training

We trained our model using down-sampled aerial imagery. We separated 90% of aerial imagery for use in training, and 10% for use in validating the trained model, repeating this process to create a 10-fold validation system wherein we iteratively trained and tested on each fold to verify that no set of images was having an undue influence on the model (S1 Fig). We tested our model's classification performance by applying it to very high-resolution satellite imagery. Manually-annotated satellite imagery, in which whales and empty ocean were identified by expert annotators, was used as the standard against which the CNN's performance was compared. It is important to note that our model was naive to 'real' satellite imagery and was applied without further refinement following its development using the down-sampled aerial training and testing dataset.

We implemented our CNN using the Pytorch framework [43], which makes it easy to implement, train, and adapt a model, and tested three different architectures: ResNet (using 18-, 34-, and 152-layer models) [44] and DenseNet [45]. These models are all widely-used and have performed well in various competitions. Each network takes as input a small image and outputs a vector of two elements, which represent the probability of the input image containing or not containing a whale, respectively. All code (S1–S6 Files), required software packages (S2 Table), and details on hardware used (S7 File) are included in the supplementary materials. Each model was pre-trained on the ImageNet dataset [46] consisting of 1.28 million training images of 1,000 different classes (e.g. ‘house,’ ‘spider,’ ‘fire’). We modify the last layer of this model to train with our data, i.e. having only 2 classes as opposed to 1,000. Pre-training the model on ImageNet has been shown to reduce overfitting and training time [46].

Whales are inherently rare in imagery and the training architecture takes only small batches (n = 4 to n = 32) of images at a time, so we risk having the model examine only images of water much of the time. As this may impair the ability of the model to learn, we implemented a weighted random sampler that increases the probability that a whale image will appear in any given batch in proportion to the number of whale images in the overall training set (S4 and S5 Files). Users can set several hyperparameters and we experimented primarily with the learning rate which governs how new information is weighted against older information. Higher learning rates down-weight older information relative to new information; if the learning rate is too high, the model will disregard previous whales it has seen in favor of the characteristics of the most recent whale it has encountered. Conversely, if the learning rate is too low, and the model is reluctant to incorporate new information. We used the set of images that were withheld from training to test the performance of each model. False positives and false negatives for each epoch were used to further tune model parameters and retrain models.

To demonstrate the value of the CNN approach, we also trained a ridge regression model (*α* = 1) and C-Support Vector Classifier model (C-SVC; *C* = 1) [47] using a reduced training set (retaining all whale images but randomly selecting an equal number of water images) and the same testing set, and implemented through Scikit-learn ([48]; S8 File). These methods, in contrast to the CNN approach, require an additional feature-extraction step implemented using a histogram of oriented gradients approach.

## Results

**Fig 3:**
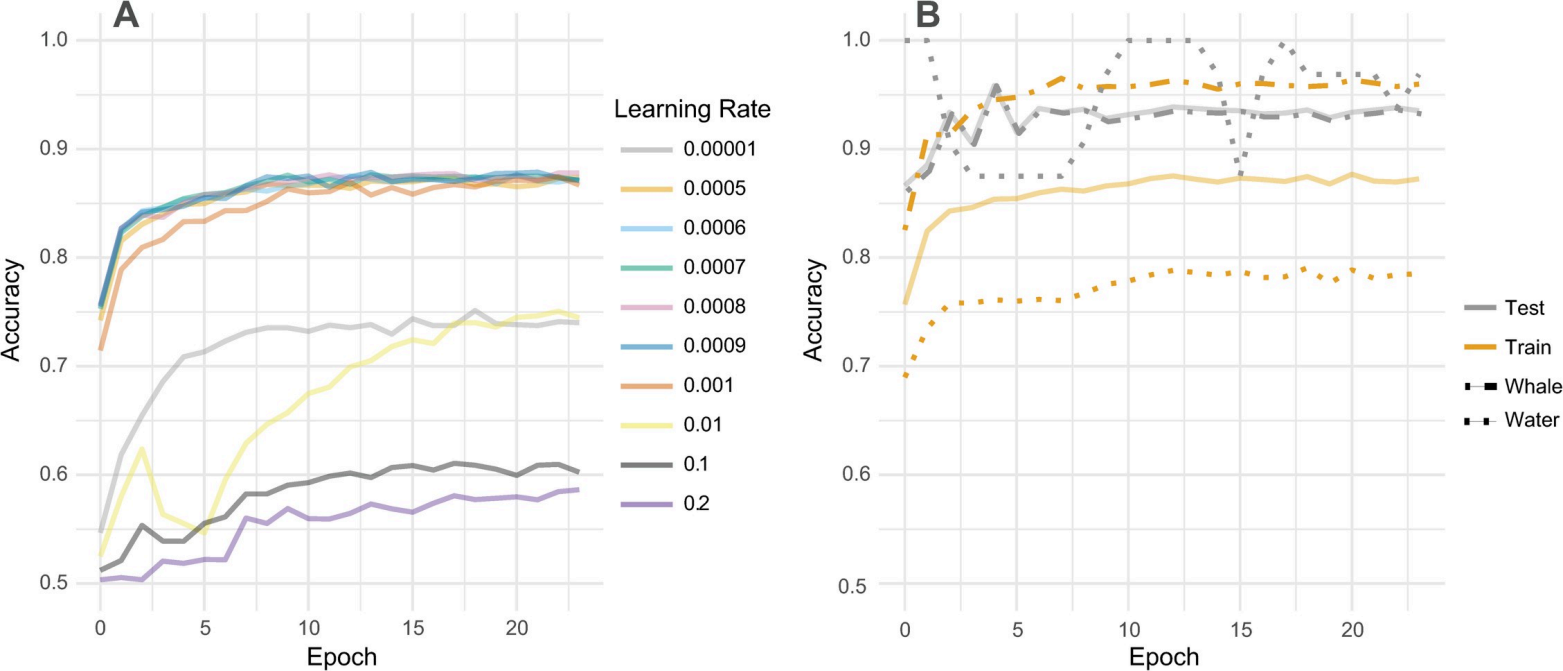
Effect of learning rate on performance. (A) Each model was trained using several different learning rates, which affected their performance on the training dataset (S9 File). (B) The best model weights came from the 12^th^ epoch of a ResNet-152 model with a learning rate of 0.0006 (S10 File). In both training and testing phases, solid lines show overall performance, while dashed and dotted lines show the accuracy of the individual classes. With a step-size of 7, the learning rate begins to decay at the 7^th^ epoch, and the accuracy begins to level out.

Image tiling took approximately 15 seconds per km^2^ though the precise timing is sensitive to the specific characteristics of the computing resources available. Model training time varied among CNN model architectures from approximately one hour (ResNet-18) to nearly 7 hours (DenseNet) for the full training set on our hardware (S7 File). We trained all models for 24 epochs and tested the model weights of each epoch against our test set of satellite images. Our best model trained for 9 epochs and used a learning rate set at 0.0006, with a step size (a parameter that allows the learning rate to decay after a certain number of epochs) of seven epochs, and a momentum of 0.9 (Table 1). We found that a higher learning rate often led to little learning, wherein the model would perform only slightly better than random chance on the training dataset (Fig 3). On the other hand, a model with a very low learning rate of 0.00001 learned very slowly During the training phase of the ten-fold validation, all folds trained along a similar trajectory.

**Table 1:** Results of variation in learning rate (LR) for ResNet-152.

Learning rate	Precision	Recall
LR = 0.00001	1.000	0.722
LR = 0.0005	1.000	0.797
**LR = 0.0006**	**1.000**	**0.937**
LR = 0.0007	1.000	0.860
LR = 0.0008	1.000	0.908
LR = 0.0009	1.000	0.895
LR = 0.001	1.000	0.915
LR = 0.01	0.996	0.707
LR = 0.1	0.987	0.898
LR = 0.2	0.986	0.950

The standard measurement of performance in this case is precision (the percent of positives [model-classified as water] that are true positives [manually-labeled as water]), and recall (the percent of manually-labeled water images that were found by the model). Precision was closely consistent among folds, ranging from 0.99 to 1.00 (that is, nearly everything classified as water was actually water such that few or no whales were misclassified), with higher variation in recall (0.33 to 1.00). This suggests that there was some variation among the training images in the folds that was affecting model performance, though most of this variation falls in only folds 1 and 10 (Table 2; S1 Fig). In particular, fold 10 had very poor performance, likely due to the fact that the test images in this fold had much rougher sea conditions than in the other scenes. Given that this fold was trained only on calmer conditions, it is not surprising that the edges and contrast of these rougher images could be mis-classified as whales (S2 Fig).

**Table 2:** Results at the final epoch of 10-fold validation.

Aerial test fold	N water training	N whale training	N water test	N whale test	Precision	Recall
**1**	11,076	207	1,230	23	0.996	0.616
**2**	11,076	207	1,230	23	1.000	0.988
**3**	11,076	207	1,230	23	0.993	0.994
**4**	11,076	208	1,230	23	0.999	0.999
**5**	11,076	208	1,230	23	0.999	0.989
**6**	11,076	207	1,230	23	1.000	0.996
**7**	11,076	207	1,230	23	1.000	0.998
**8**	11,076	207	1,230	23	1.000	1.000
**9**	11,076	207	1,230	23	0.955	1.000
**10**	12,306	206	1,236	24	0.998	0.322

**Fig 4:**
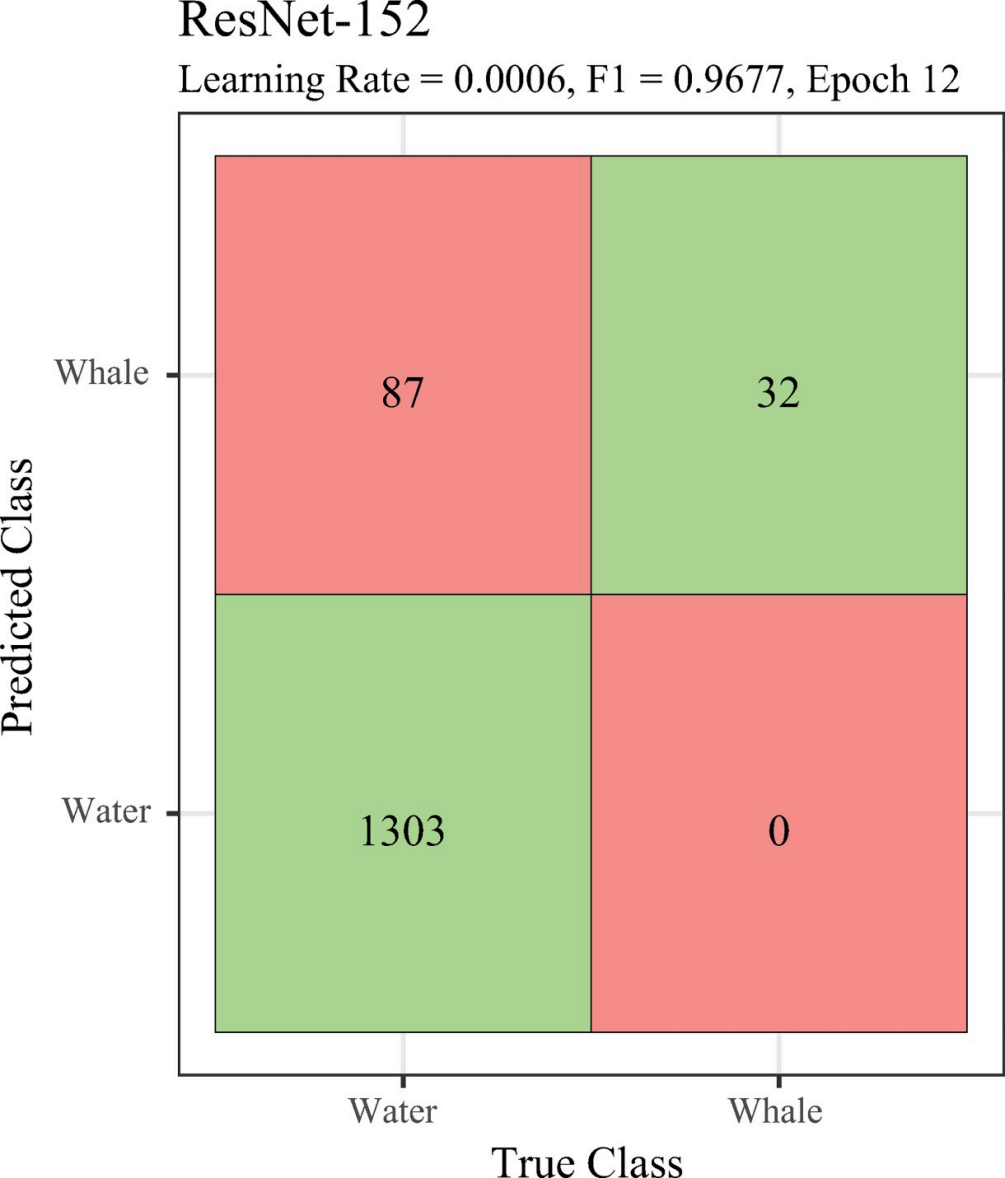
Model performance. Confusion matrix for the best model–ResNet-152. Full table of performance for all models available in S11 File.

All CNN model architectures succeeded in finding all or nearly all whales in our test set, and correctly classified nearly all water images, with the best model performance resulting in an F1 score, $\frac{2*precision*recall}{precision+recall}$, of 0.968 (Table 3; Fig 4). By contrast, the more traditional classification methods (ridge regression and C-SVC) performed more poorly than any of the CNN models, finding only 88% of the whales in the test set (Ridge regression: precision = 0.996, recall = 0.678, F1 = 0.807; C-SVC: precision = 0.995, recall = 0.632, F1 = 0.773). Modifying the parameter α in ridge regression determines the weight given to the residual sum of squares and the sum of square coefficients. The outcome for *α* = 0 was F1 = 0.827 versus 0.804 for *α* = 10,000, suggesting that for this problem, ridge regression is little better than a simple linear regression. For C-SVC, *C* is a penalty term that controls the prioritization of classification accuracy versus smooth boundaries. Similar to ridge regression, tuning this parameter did not significantly improve the model (*C* = 0.001, F1 = 0.773; *C* = 10,000, F1 = 0.799).

**Table 3:** Performance of different model types.

Model	Precision	Recall	F1 Score	Epoch	LR
**ResNet-18**	1.000	0.932	0.965	24	0.0009
**ResNet-34**	1.000	0.932	0.965	20	0.0009
**ResNet-152**	**1.000**	**0.937**	**0.968**	**9**	**0.0006**
**DenseNet**	0.999	0.922	0.959	5	0.001

## Discussion

Here we describe a functioning pipeline for identifying whales in high-resolution satellite imagery that can be immediately employed to reduce the time required to complete large-extent surveys. All ResNet versions performed well, correctly classifying all whales and at this stage we recommend ResNet-152 for its high accuracy, correctly classifying all whales and mis-classifying only 87 of 1390 water images in WorldView-3 imagery for a false positive rate of about 6.1% (Table 3; Fig 4). DenseNet also performed well but did not match the success of ResNet. Neither C-SVC nor ridge regression matched the performance of any of the CNN models, likely reflecting the subtle appearance of whales in imagery and the degree of variation among whales and among scenes involving different water conditions and suggesting that neither is well-suited to this task. In this case the F1 score demonstrates the quality of the ResNet-152 model, though these scores can be misleading. In our case it is more important to maximize precision than recall, as false positives can easily be thrown out, but examining all false negatives requires the same amount of time as manually annotating, defeating the purpose of the automated approach. Importantly, the assignment of one class or another as “positive” or “negative” is arbitrary. Our model considered water as the positive case; were it the other way around, it would be more important to maximize recall.

We have deployed multiple model architectures here and received promising results with several of them, yet future development in deep learning will likely outstrip their performance. We have tuned our models to our particular problem and dataset, but the optimal parameters for our dataset are not universal. For example, ResNet-18 required a full 24 epochs of training to reach model weights that performed well on satellite imagery. ResNet-152, a “deeper” and “wider” network, arrived at its best weights after only 9 epochs (Table 3). In this case, both were trained with the same set of experimental learning rates, but this and other parameters could be tuned to allow for longer or shorter training. In the case of ResNet-152, the model weights at the 24^th^ epoch demonstrated that the model had overfit the training data slightly, and this can easily change with a different training set, especially one of different size.

Like most machine-learning applications, the model could be iteratively improved with the addition of correctly classified and verified whales from future imagery, and our current classification accuracy therefore represents a lower bound on the potential for satellite imagery to aid in cetacean surveys. We believe that this method is an improvement over the previous classification methods employed by Fretwell et al. [24] which, while successful, will likely be sensitive to differences in ocean color and turbidity and less robust to the size of different species. Our method differs from Fretwell et al. [24] in several respects, not only in regard to the classification method but also with respect to the underlying data used (that is, red, green, and blue bands only), and one avenue for further research may be to explore the benefit of using additional spectral bands for classification. Despite the demonstrated feasibility of automated classification of whales in satellite imagery, barriers remain to broad adoption. Most significant is the paucity of open-water imagery available in DigitalGlobe’s archive (and the lack of similar-resolution sensors from other providers), which reflects that imagery is not collected continuously but is instead targeted within high-demand regions or in response to specific orders from customers. Hopefully, more interest in using satellite imagery for marine mammal surveys will facilitate the expansion of open-water imagery available within the catalog.

The current pricing structure for very high-resolution imagery would likely prevent many research applications from pursuing projects at basin-wide scales, but alternative pricing for non-profit organizations and education users is available. While it is difficult to estimate the cost of field surveys given the differing logistics based on time and region, Abileah [49] suggests costs should be similar to aerial surveys and a substantial savings in more remote areas. Satellite tasking logistics can make imagery acquisition in some locations (e.g., high latitudes) challenging, though plans for larger satellite constellations will ameliorate many of these limitations over time. Encouraging the collection of open-water imagery in areas of interest and in areas of low competition is the first step in moving imagery-based methods to broad applicability. In the meantime, the pooling of aerial photographs with known ground-sample distance by different research groups could result in a more robust training image set, and users with large catalogs of aerial imagery for their specific taxa and regions could create bespoke local training sets to better classify cetaceans in their region of interest.

**Fig 5:**
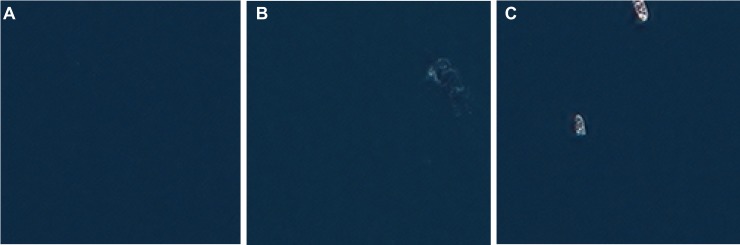
Example images. Open water (A), Southern right whale (B), whale-watching boat (C). Satellite imagery published under a CC BY license, with permission from the DigitalGlobe Foundation, original copyright 2014.

Given that our model is trained exclusively on minke whales, the smallest of the baleen whales, including more aerial photography of larger whale species would likely further improve performance. That said, it performed surprisingly well on whales that can reach double the size of a minke whale. The code could easily be modified to create overlapping tiles, which would eliminate problems arising from whales bisected by neighboring tiles (S1 File). The addition of further classes representing objects such as boats, large ships, land, and rocks at the surface in the imagery would also help minimize the number of false positives in a cetacean survey (Fig 5). A greater number of classes would allow the model to more accurately classify objects that don’t fit neatly into the water or whale category; currently those objects are forced into one of the existing categories even if they are a poor visual match. Such classes could be fine-tuned to the application at hand with training images added for the particular conditions found in a region, such as peculiar boat shapes or floating rafts of detritus. Further classification to the species level is theoretically possible but only with a much more robust training set. Work on manual species classification from satellite imagery shows promise but also indicates that some species are more readily identifiable than others [25].

Limitations inherent to this method are not dissimilar to those faced by any other survey method. Challenging sea-state conditions are common to boat- and aerial-based surveying [50–53] because waves and sea spray create a lot of “noise” which makes it difficult to separate a whale from the surrounding water. We did not attempt to use satellite imagery to locate whales in choppy water. It is worth noting, however, that aerial or satellite methods would likely have more success than surface-level observations as the orthogonal view allows the observer to see at least partially through the water. While we have not developed a definitive threshold for sea state, we expect that observations above Beaufort-4 observations would be difficult given widespread whitecaps at the surface. Below Beaufort-4, the size of “noisy” elements on the water below Beaufort-4 are likely to be in the range of a single pixel (31 x 31 cm) and discrimination of whales feasible. Cloud cover is also a controlling factor, and future applications will need to pair this detection pipeline with appropriate statistical models for non-detection [54].

Aside from environmental challenges, there are several satellites currently in orbit that could be used for cetacean surveying, such as previous iterations of WorldView and Pleiades. While this trained model may be robust to differences in spatial resolution among the various sensors available, we did not test imagery from other sensors. Sensor-specific models could easily be trained, as the aerial imagery can be down-sampled to any resolution desired. Scaling this method to process larger volumes of imagery will be manageable for an individual user for small areas, but once the spatial and temporal scope increases, tailored cyberinfrastructure (such as is underway with the ICEBERG project; https://iceberg-project.github.io) will be required to handle both the storage and transmission of imagery to a computing cluster [55].

This method could be used to improve cetacean research in several different ways. It provides a means of viewing and monitoring areas that are far from ports or are hazardous to access, such as polar regions, remote island chains, or open ocean. Moreover, it provides the potential to monitor these areas at a daily time scale, cloud-cover permitting. With enough imagery, it could be used to monitor the arrival of migrating species or examine fine-scale changes in foraging activity. Long-term studies on whale feeding and breeding grounds have provided critical information on the ecology and behavior of these animals but are poorly suited to answer basin-scale questions for species that range widely both within and among seasons. The arrival time of migrating whales at traditional feeding grounds, for example, has been used to understand links between habitat use and local environmental conditions [56], but is unable to illuminate the existence of unmonitored areas that may serve as alternative feeding grounds. With the ability to rapidly and automatically detect whales in satellite imagery, boat or aerial surveys become valuable as ground-truthing rather than as the sole source of data on whale abundance and distribution, and researchers intent on instrumenting individuals or collecting individual-level data may be able to more accurately target their effort, saving time and expense. While far from a total replacement for other survey modalities, this method has promise to improve current survey methodology for large whales, increase the temporal resolution of surveys, expand the ocean surface area surveyed, minimize human risk, and increase the rate of data acquisition.

## Supporting information

S1 Table Satellite imagery. We acquired imagery from Digital Globe’s WorldView-3 sensor via the Digital Globe Foundation. See https://discover.digitalglobe.com/ for details on individual scenes and a preview.
(PDF)

S2 Table Python packages. The code requires packages for Python 3 to be pre-installed.
(PDF)

S1 File Image tiling code. (TXT)

S2 File Image resizing code. (TXT)

S3 File Model testing code. (TXT)

S4 File Code utilities. (TXT)

S5 File Model training code. (TXT)

S6 File Model utility. (TXT)

S7 File Details on hardware. (PDF)

S8 File Ridge regression/SVM code. (TXT)

S9 File Training accuracy. Accuracy and loss at different learning rates, used to create Fig 3A.
(CSV)

S10 File Best model results. Results at the training and testing phase for a ResNet-152 model. Used to create Fig 3B.
(CSV)

S11 File Model outcomes. Classification outcomes for each model tested.
(CSV)

S1 Fig Accuracy, loss, and testing results on validation folds. Training and testing results for 10-fold validation: accuracy and loss for each fold during the training process (A). The precision and recall for each fold (B).
(PDF)

S2 Fig Sea conditions potentially affecting 10-fold validation. (PDF)
