# Supplementary material for: Aerial-trained deep learning networks for surveying cetaceans from satellite imagery
Source: PLoS One. 2019 Oct 1;14(10):e0212532. doi: 10.1371/journal.pone.0212532 (PMC6772036; doi:10.1371/journal.pone.0212532)
Supplement: S1 Table — We acquired imagery from Digital Globe’s WorldView-3 sensor via the Digital Globe Foundation. See https://discover.digitalglobe.com/ for details on individual scenes and a preview. (PDF) [file pone.0212532.s001.pdf]

S1 Table. Satellite Imagery.

We acquired imagery from Digital Globe's WorldView-3 sensor via the Digital Globe Foundation.

See https://discover.digitalglobe.com/ for details on individual scenes and a preview.

Scene ID	Location	Date
1040010003121A00	Peninsula Valdes, Argentina	14 Oct. 2014
1040010029924200	Maui, Hawaii, USA	13 Feb. 2013
