# Supplementary material for: Aerial-trained deep learning networks for surveying cetaceans from satellite imagery
Source: PLoS One. 2019 Oct 1;14(10):e0212532. doi: 10.1371/journal.pone.0212532 (PMC6772036; doi:10.1371/journal.pone.0212532)
Supplement: S2 Table — The code requires packages for Python 3 to be pre-installed. (PDF) [file pone.0212532.s002.pdf]

S2 Table. Python packages.

The code requires packages for Python 3 to be pre-installed.

Most can be installed via *pip install* <> or *conda install* <>.
For PyTorch, go to https://pytorch.org/get-started/locally/
to determine which version to install.

**Package**	**version**
Conda	4.5.8
Dominate	2.3.1
Numpy	1.15.2
Pandas	0.24.2
Pillow	5.1.0
Scikit-image	0.14.0
Scikit-learn	0.19.1
Scipy	1.1.0
Torch	0.4.0
Torchfile	0.1.0
Torchvision	0.2.1
