# Supplementary material for: Aerial-trained deep learning networks for surveying cetaceans from satellite imagery
Source: PLoS One. 2019 Oct 1;14(10):e0212532. doi: 10.1371/journal.pone.0212532 (PMC6772036; doi:10.1371/journal.pone.0212532)
Supplement: S7 File — (PDF) [file pone.0212532.s009.pdf]

Supplement S7 File

S7 File. Hardware details.

All image processing and model training and validation was completed on the SeaWulf cluster at the Institute for Advanced Computational Science (Stony Brook University, Stony Brook, NY), using GPU nodes equipped with Nvidia K80 GPUs @ 2.91 DP Teraflops. Other CUDA-enabled GPUs would also be sufficient.
