# Supplementary material for: Aerial-trained deep learning networks for surveying cetaceans from satellite imagery
Source: PLoS One. 2019 Oct 1;14(10):e0212532. doi: 10.1371/journal.pone.0212532 (PMC6772036; doi:10.1371/journal.pone.0212532)
Supplement: S1 Fig — Training and testing results for 10-fold validation: accuracy and loss for each fold during the training process (A). The precision and recall for each fold (B). (PDF) [file pone.0212532.s014.pdf]

**S1 Fig. Accuracy, loss, and testing results on validation folds.**

Training and testing results for 10-fold validation: accuracy and loss for each fold during the training process (A). The precision and recall for each fold (B).

**A**

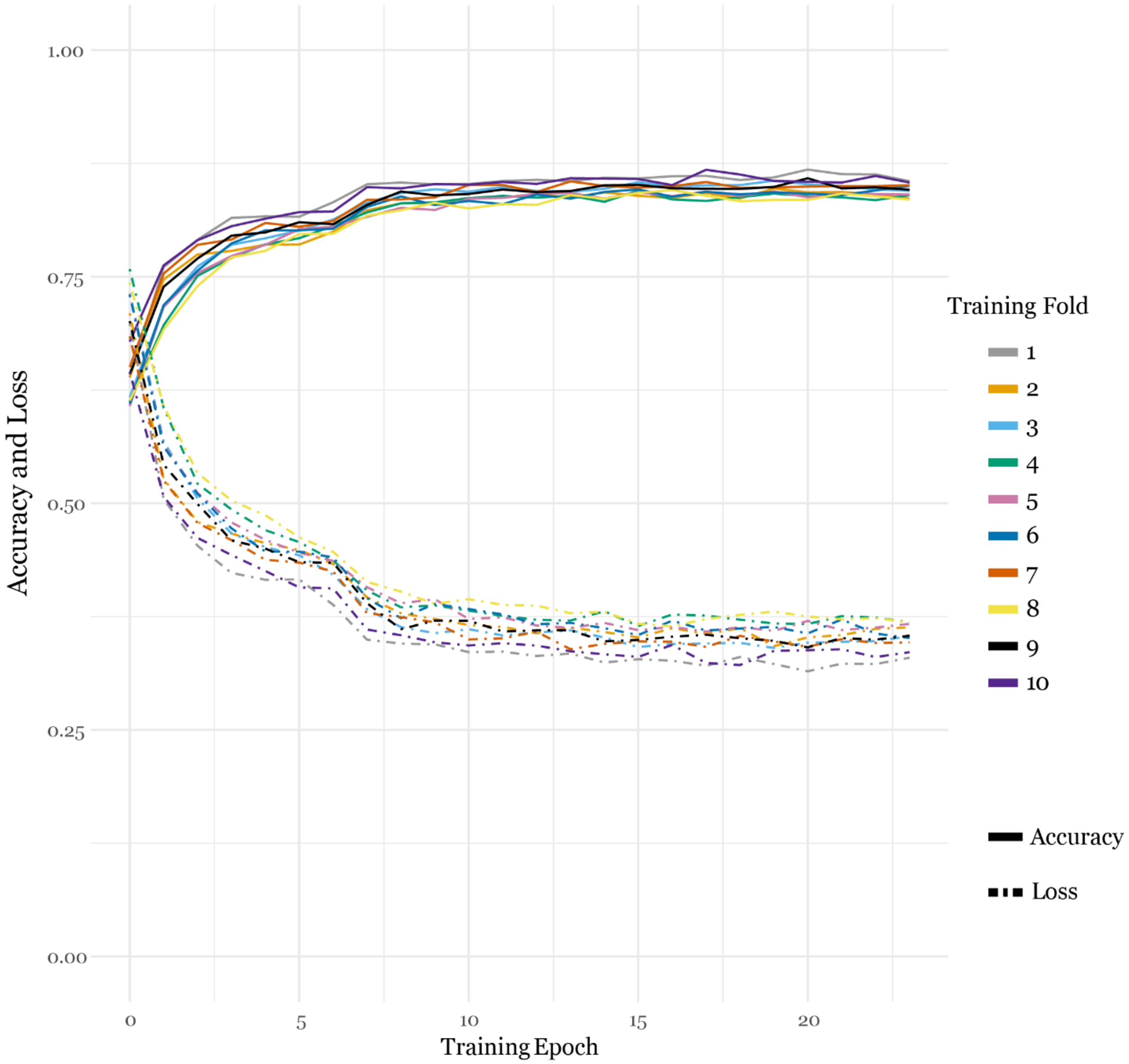

**B**

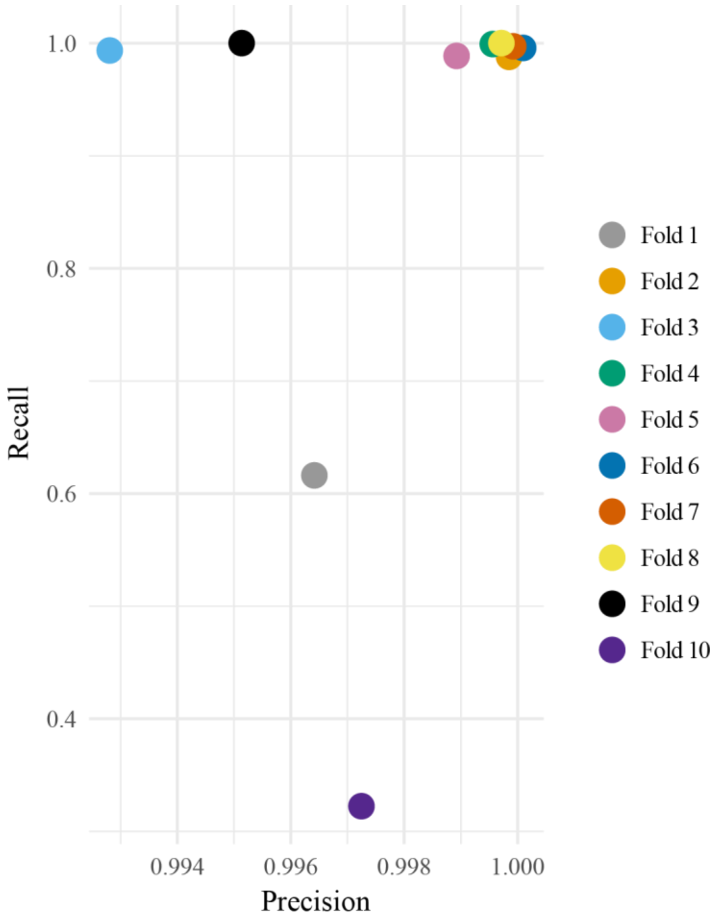
1.0
0.8
0.6
0.4
Recall
0.994
0.996
0.998
1.000
Precision
Fold 1
Fold 2
Fold 3
Fold 4
Fold 5
Fold 6
Fold 7
Fold 8
Fold 9
Fold 10
