# Supplementary material for: Aerial-trained deep learning networks for surveying cetaceans from satellite imagery
Source: PLoS One. 2019 Oct 1;14(10):e0212532. doi: 10.1371/journal.pone.0212532 (PMC6772036; doi:10.1371/journal.pone.0212532)
Supplement: S2 Fig — (PDF) [file pone.0212532.s015.pdf]

S2 Fig. Confusion matrices for model versions.

Confusion matrices for each trained model – combinations of model type (ResNet-18, ResNet-34, ResNet -152, DenseNet) and learning rate (LR=0.2, 0.1, 0.01, 0.001, 0.0009). See Table 2 in-text.

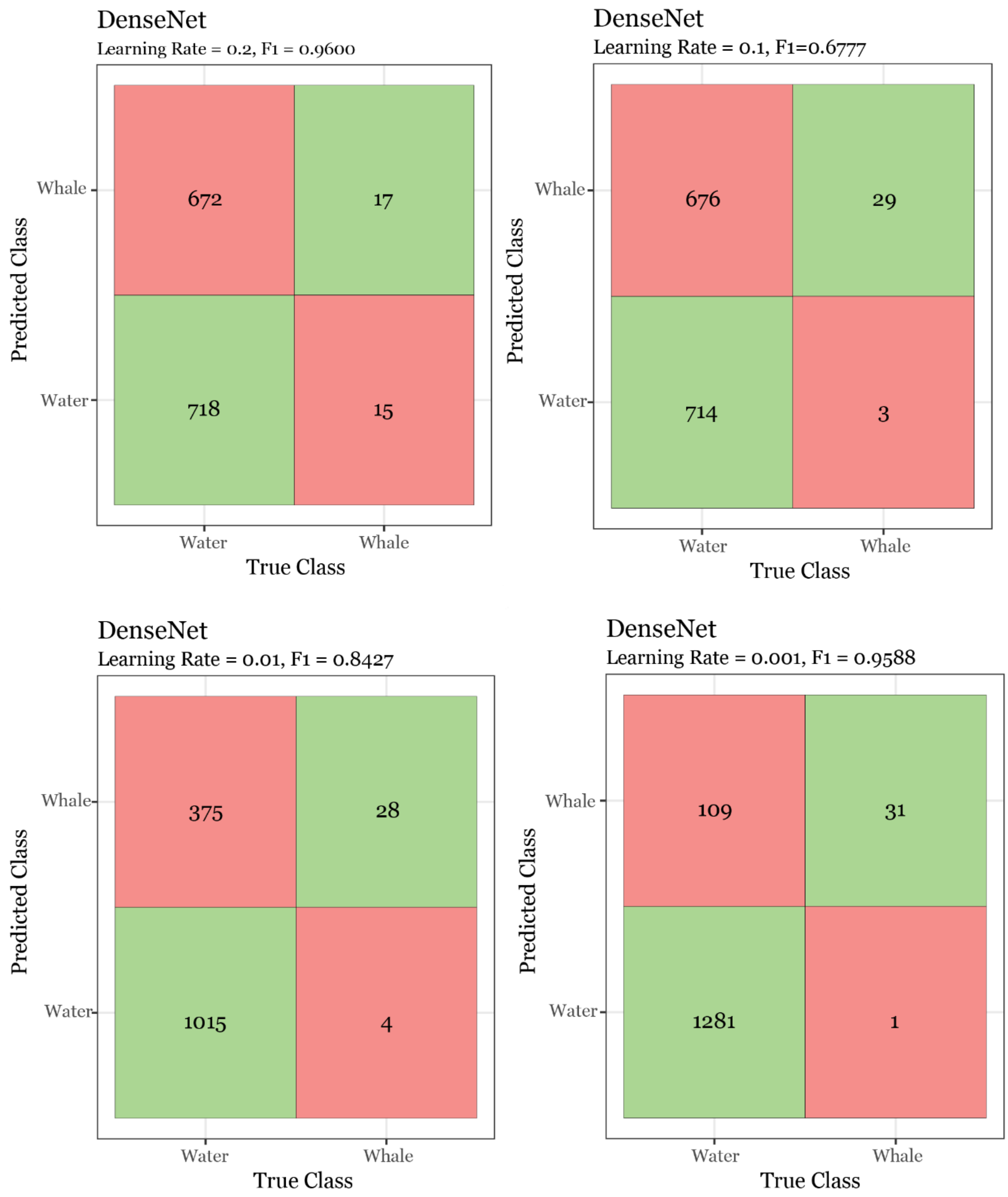

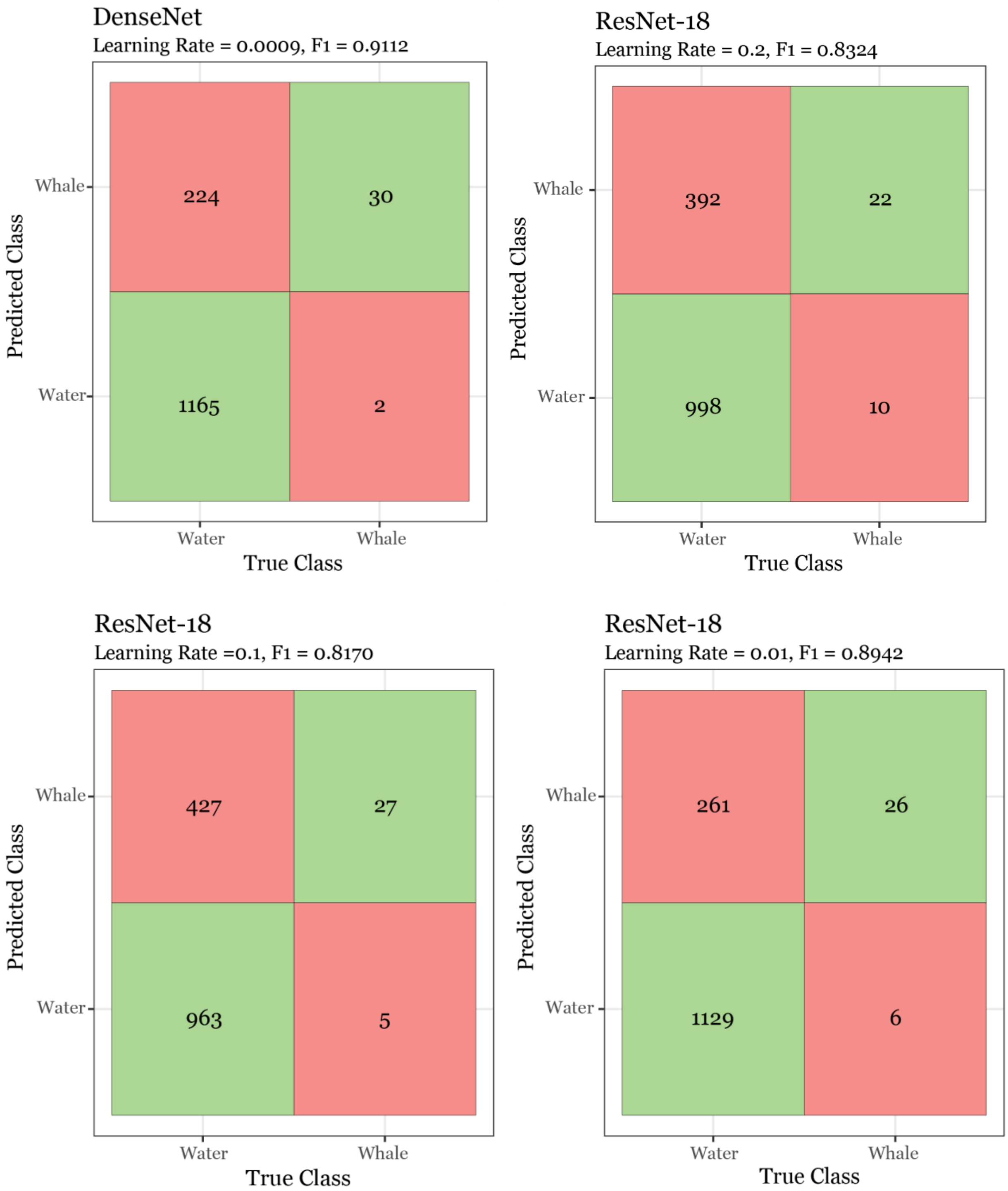
DenseNet
Learning Rate = 0.0009, F1 = 0.9112
Predicted Class
Whale
Water
224
30
1165
2
Water
Whale
True Class
ResNet-18
Learning Rate = 0.2, F1 = 0.8324
Predicted Class
Whale
Water
392
22
998
10
Water
Whale
True Class
ResNet-18
Learning Rate =0.1, F1 = 0.8170
Predicted Class
Whale
Water
427
27
963
5
Water
Whale
True Class
ResNet-18
Learning Rate = 0.01, F1 = 0.8942
Predicted Class
Whale
Water
261
26
1129
6
Water
Whale
True Class

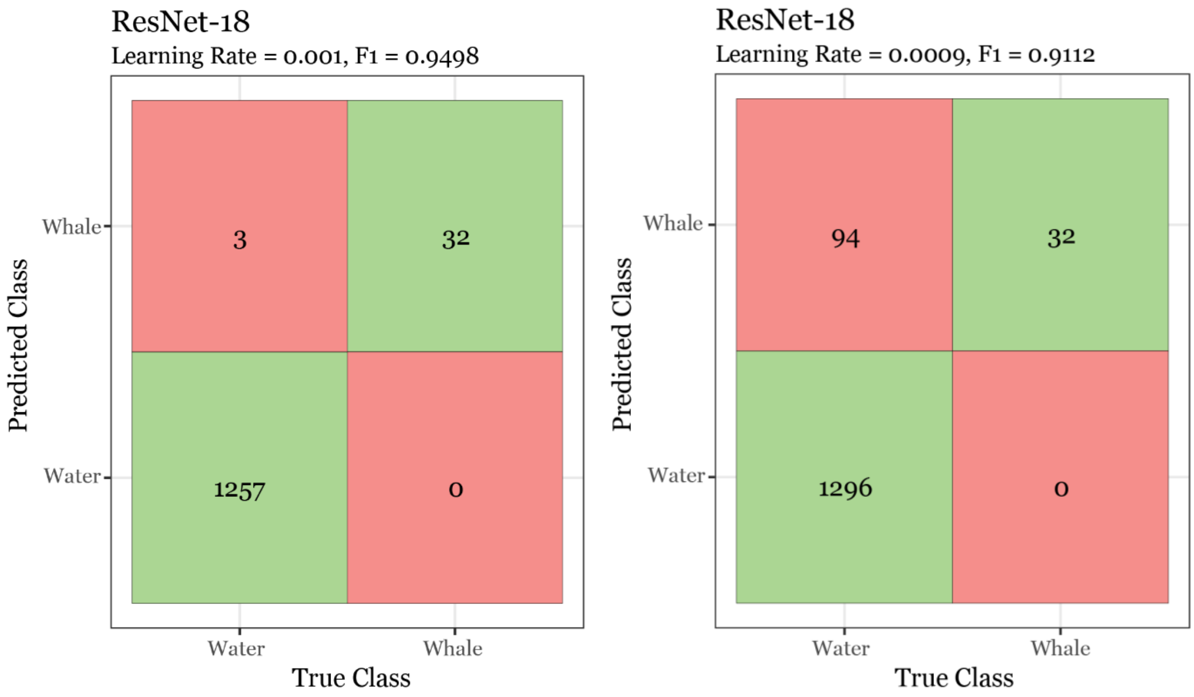
ResNet-18
Learning Rate = 0.001, F1 = 0.9498
Predicted Class
Whale
Water
3
32
1257
0
Water
Whale
True Class
ResNet-18
Learning Rate = 0.0009, F1 = 0.9112
Predicted Class
Whale
Water
94
32
1296
0
Water
Whale
True Class

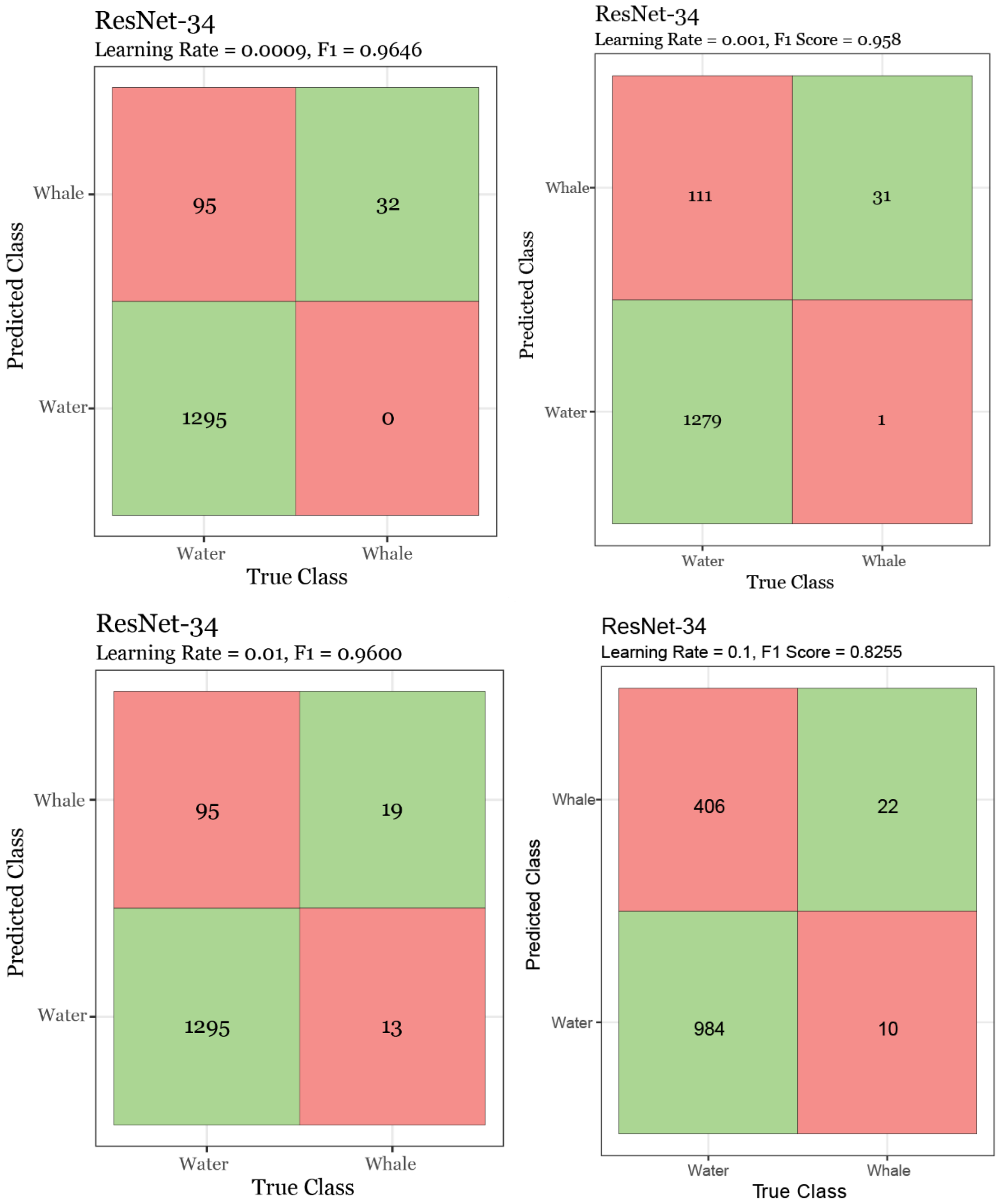
ResNet-34
Learning Rate = 0.0009, F1 = 0.9646
Predicted Class
Whale
Water
95
32
1295
0
Water
Whale
True Class
ResNet-34
Learning Rate = 0.001, F1 Score = 0.958
Predicted Class
Whale
Water
111
31
1279
1
Water
Whale
True Class
ResNet-34
Learning Rate = 0.01, F1 = 0.9600
Predicted Class
Whale
Water
95
19
1295
13
Water
Whale
True Class
ResNet-34
Learning Rate = 0.1, F1 Score = 0.8255
Predicted Class
Whale
Water
406
22
984
10
Water
Whale
True Class

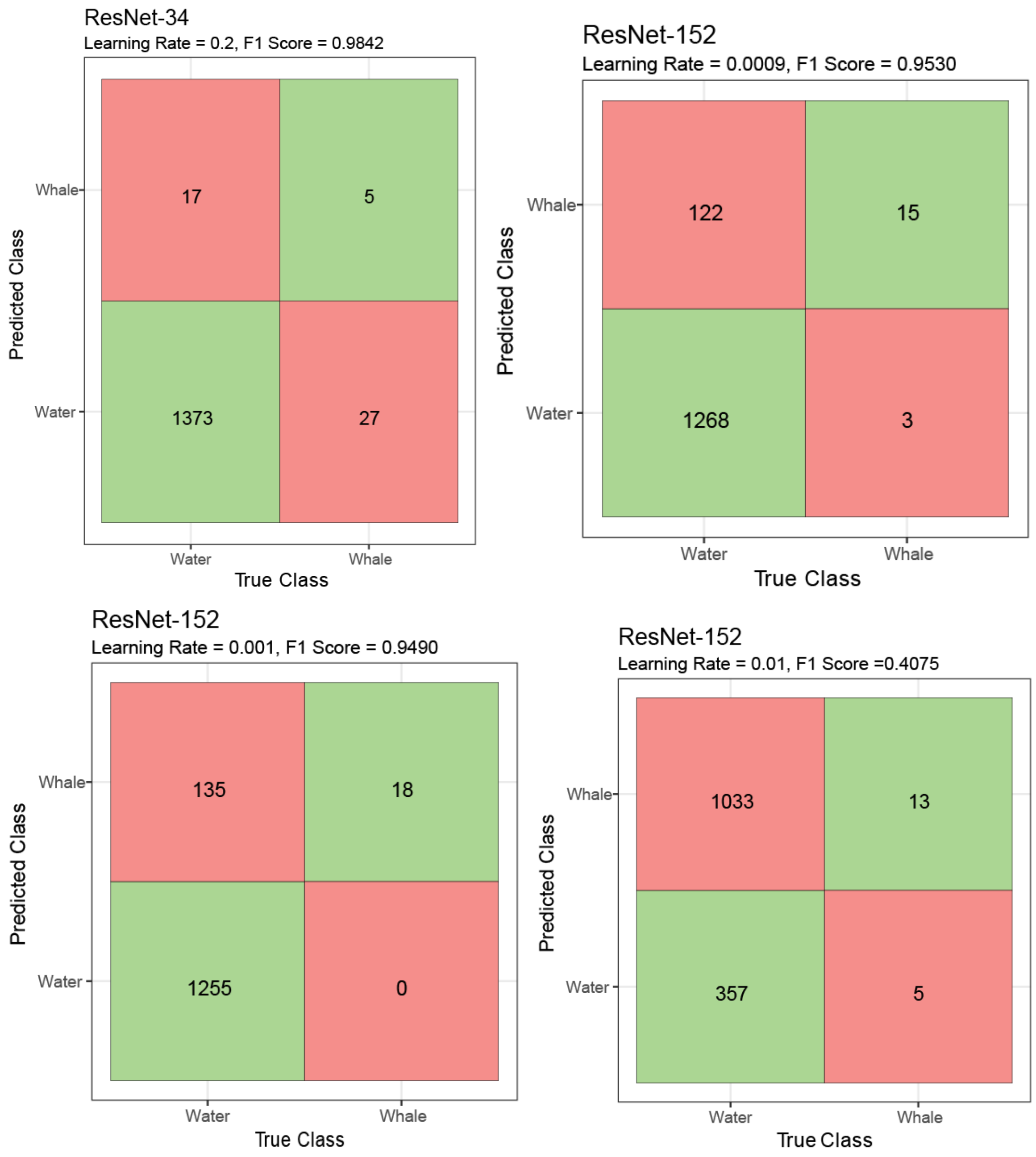
ResNet-34
Learning Rate = 0.2, F1 Score = 0.9842
Predicted Class
Whale
Water
17
5
1373
27
Water
Whale
True Class
ResNet-152
Learning Rate = 0.0009, F1 Score = 0.9530
Predicted Class
Whale
Water
122
15
1268
3
Water
Whale
True Class
ResNet-152
Learning Rate = 0.001, F1 Score = 0.9490
Predicted Class
Whale
Water
135
18
1255
0
Water
Whale
True Class
ResNet-152
Learning Rate = 0.01, F1 Score =0.4075
Predicted Class
Whale
Water
1033
13
357
5
Water
Whale
True Class

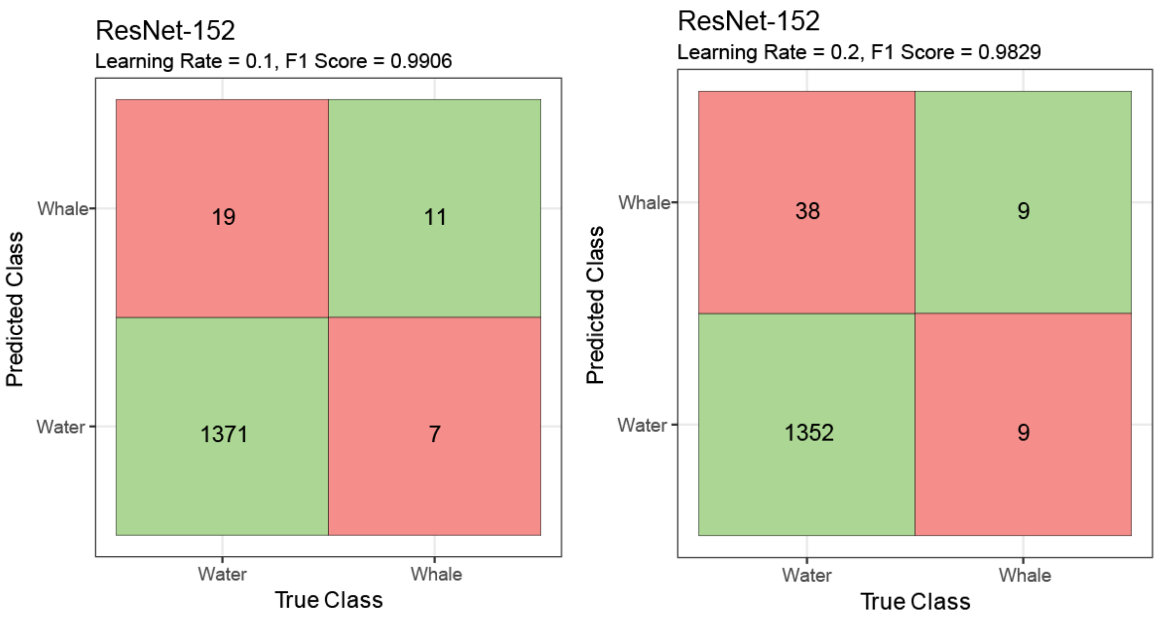
ResNet-152
Learning Rate = 0.1, F1 Score = 0.9906
Predicted Class
Whale
Water
19
11
1371
7
Water
Whale
True Class
ResNet-152
Learning Rate = 0.2, F1 Score = 0.9829
Predicted Class
Whale
Water
38
9
1352
9
Water
Whale
True Class
